# Supplementary material for: PJA1-mediated suppression of pyroptosis as a driver of docetaxel resistance in nasopharyngeal carcinoma
Source: Nat Commun. 2024 Jun 21;15:5300. doi: 10.1038/s41467-024-49675-2 (PMC11192944; doi:10.1038/s41467-024-49675-2)
Supplement: Supplementary file 1 — Supplementary Information [file 41467_2024_49675_MOESM1_ESM.pdf]

Supplementary Information

PJA1-mediated suppression of pyroptosis as a driver of docetaxel resistance in nasopharyngeal carcinoma

Huang et al.,

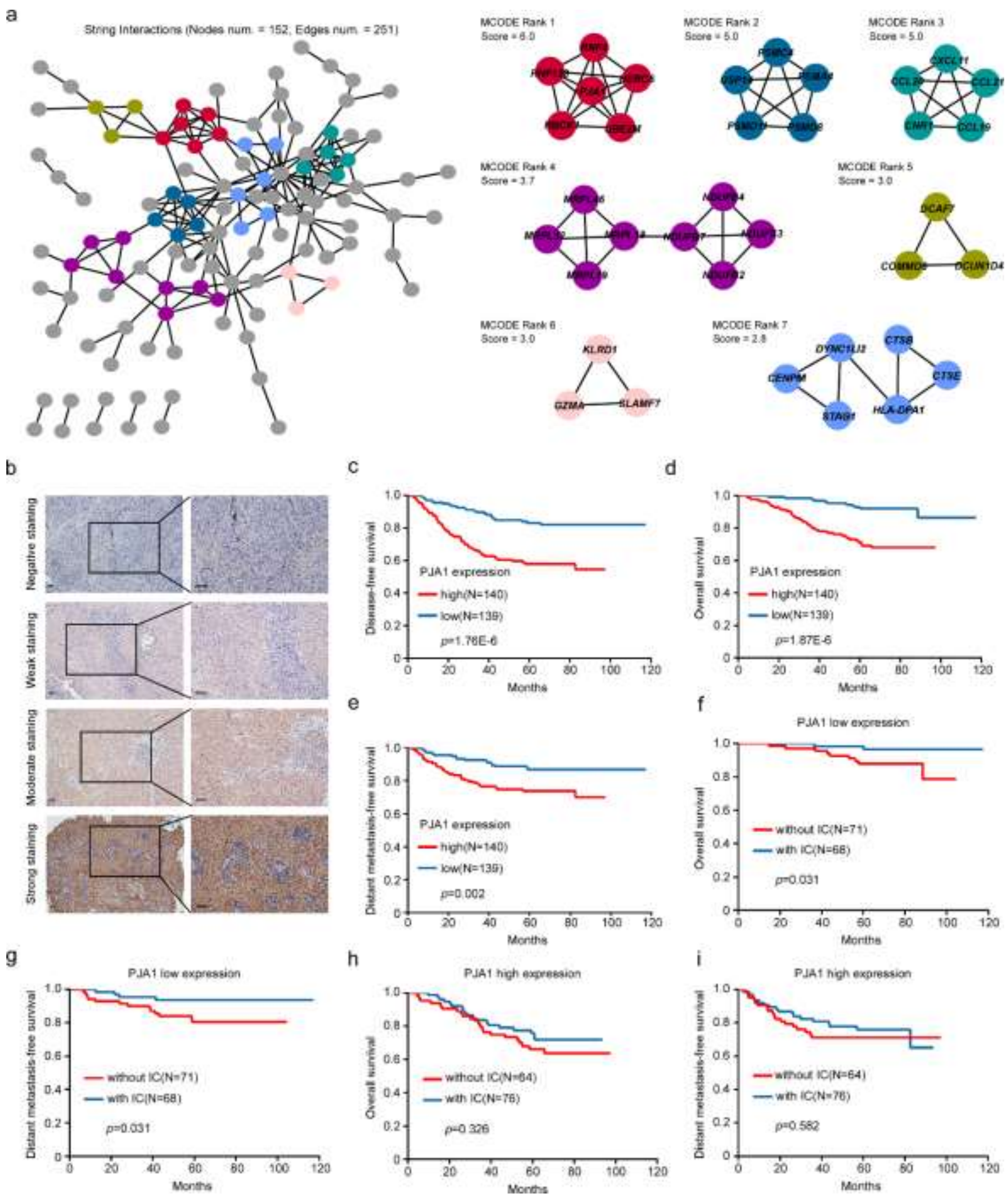

Supplementary Fig. 1 (Extended data related to Main Fig. 1)

a, left: 385 differentially expressed genes (DEGs) were used to construct the protein-protein interaction (PPI) network, which contained 152 nodes and 251 edges; Right: Module analysis of the PPI network. **b,** Representative images of IHC staining for PJA1 protein expression is graded according to the staining intensity in NPC tissues (scale bar, 50 µm). **c-e,** Kaplan–Meier analysis of disease-free survival (**c**), overall survival (**d**) and distant metastasis-free survival (**e**) in NPC patients with low PJA1 expression (n=139) or high PJA1 expression (n=140) (log-rank test). **f-g,** Kaplan–Meier analysis of overall survival (**f**) and distant metastasis-free survival (**g**) in NPC patients treated with (n=68) or without (n=71) induction chemotherapy (IC) in the low PJA1 expression group. **h-i**, Kaplan–Meier analysis of overall survival (**h**) and distant metastasis-free survival (**i**) in NPC patients treated with (n=76) or without (n=64) IC in the high PJA1 expression group.

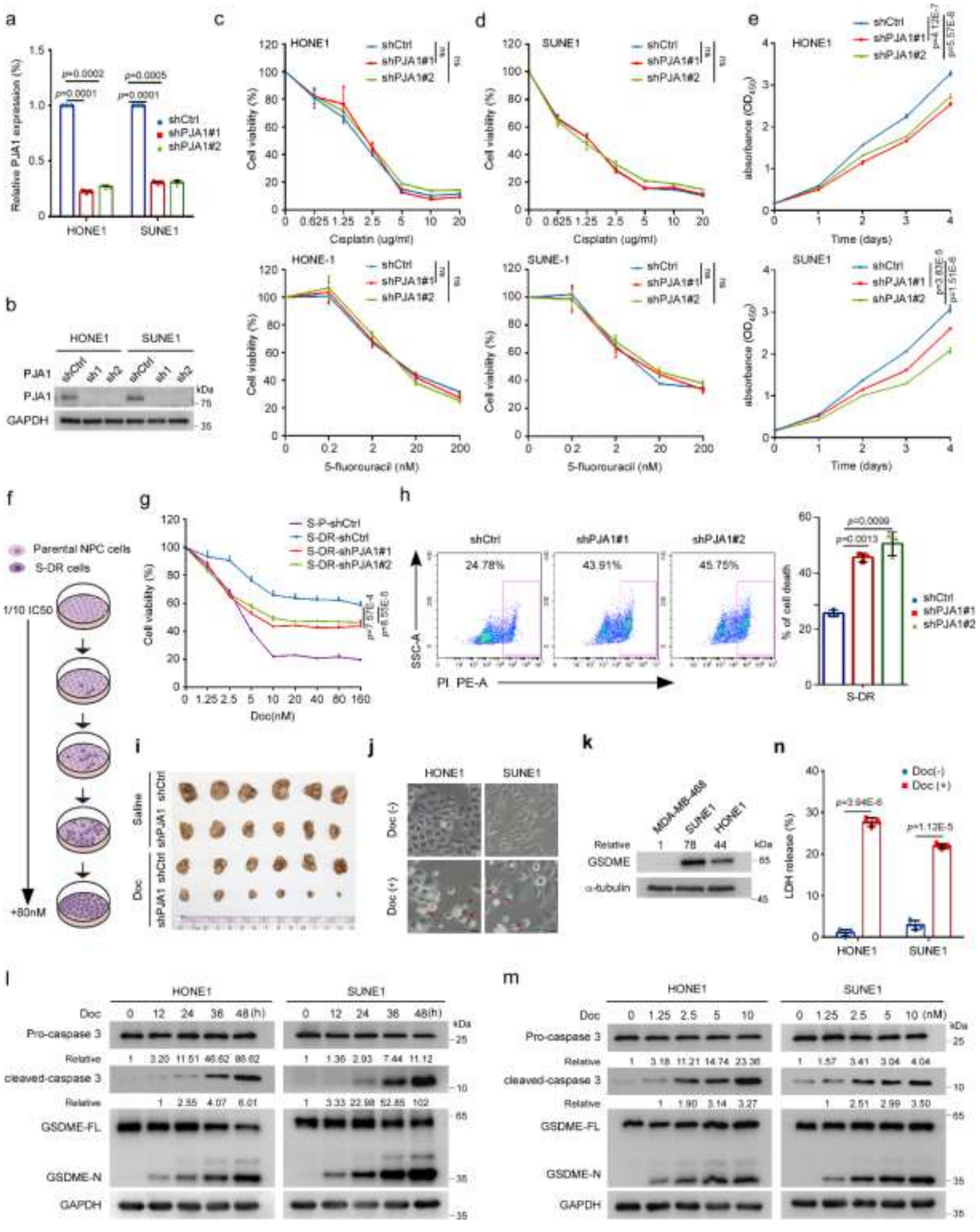

Supplementary Fig. 2 (Extended data related to Main Fig. 1)

a-b, Relative mRNA (**a**, mean ± s.d., one-way ANOVA) and protein (**b**) expression of PJA1 in NPC cells transfected with the shCtrl or sh-PJA1s plasmids. **c-d,** CCK8 assay measuring the chemosensitivity of HONE1 (**c**) and SUNE1 (**d**) cells transfected with the shCtrl or sh-PJA1s plasmids and exposed to the indicated concentrations of cisplatin and 5-fluorouracil (mean ± s.d., two-way ANOVA). **e**, CCK-8 assays in SUNE-1 and HONE-1 cells transfected with shCtrl and ShPJA1 and ShPJA2 (mean ± s.d., two-tailed unpaired t test). **f**, the workflow process of constructing a docetaxel-resistant SUNE1 cell line (S-DR cell). **g**. CCK8

assay measuring the chemosensitivity of S-DR cells transfected with the shCtrl or sh-PJA1s plasmids and exposed to the indicated concentrations of docetaxel (Doc, mean ± s.d., two-tailed unpaired t test). **h,** Flow cytometry analysis of cell death in S-DR cells transfected with the shCtrl or sh-PJA1s plasmids and exposed to Doc (10 nM) for 48h (mean ± s.d., one-way ANOVA with Dunnett's multiple comparisons test). **i,** Macroscopic images of excised tumours. **j,** Representative images of pyroptotic morphology in NPC cells exposed to docetaxel (10 nM). The red arrow indicates pyroptotic cells. Scale bar, 25 μm. **k,** GSDME protein expression in MDA-MB-468, SUNE1 and HONE1 cells measured by western blotting. **l-m,** Cleavage of caspase-3 and GSDME in NPC cells treated with docetaxel at the indicated concentrations (**l**) or for the indicated times (**m**). **n,** LDH release in NPC cells exposed to docetaxel (10 nM, mean ± s.d., two-tailed unpaired t test). N=4 (c, d, e, g), n=3(h, n) repeats from three independent experiments. Source data are provided as a Source Data file.

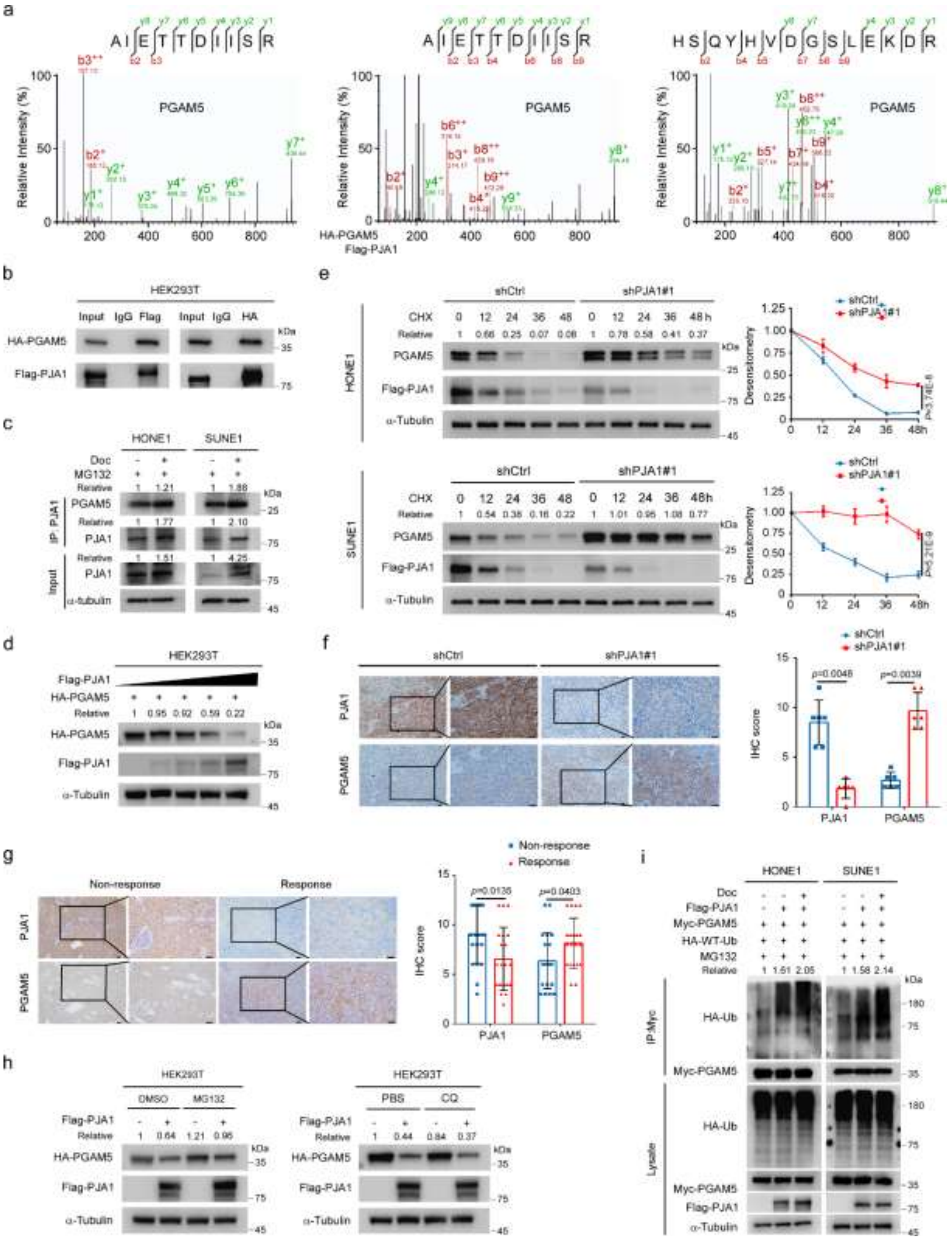

Supplementary Fig. 3 (Extended data related to Main Fig. 2)

a, PJA1 junction-specific peptides of PGAM5 were identified by immunoprecipitation with an anti-Flag antibody in SUNE1 cells overexpressing Flag-PJA1. **b,** Co-IP with an anti-Flag (top) or anti-HA (bottom) antibody revealed the exogenous association of PJA1 and PGAM5 in HEK293T cells. **c,** Co-IP with an anti-PJA1 antibody revealed the endogenous association of PJA1 and PGAM5 in NPC cells exposed to

docetaxel(10nM) or not. **d,** Protein expression of PGAM5 in HEK293T cells transfected with gradient concentrations of the Flag-PJA1 plasmids. **e,** Immunoblot (left) and corresponding greyscale analysis (right) of PGAM5 expression in NPC cells stably overexpressing PJA1 transfected with the shCtrl or sh-PJA1 plasmids after the CHX treatment (mean (n = 3) ± s.d., two-way ANOVA). **f,** Representative images of IHC staining and IHC scores for PJA1 and PGAM5 expression in excised tumours from the xenograft models which established by stably transfecting SUNE1 cells with the shCtrl or shPJA1 plasmids. Scale bars, 50 μm (mean (n = 6) ± s.d., two-tailed unpaired t test). **g,** Representative images of IHC staining and IHC scores for PJA1 and PGAM5 expression in NPC tumours with response (n=20) or non-response(n=20) to induction chemotherapy (IC) with the docetaxel-cisplatin-5-fluorouracil (TPF) regimen. Scale bars, 50 μm (mean (n = 20) ± s.d., two-tailed unpaired t test). **h,** PGAM5 protein levels in HEK293T cells transfected with the empty vector or Flag-PJA1 plasmids together with the HA-PGAM5 plasmids after treatment with MG132 and CQ. **i,** NPC cells transfected with the empty vector or Flag-PJA1 plasmid together with Myc-PGAM5 and HA-WT-Ub were subjected to denaturing IP with the indicated antibodies. N=3 (e) repeats from three independent experiments. Source data are provided as a Source Data file.

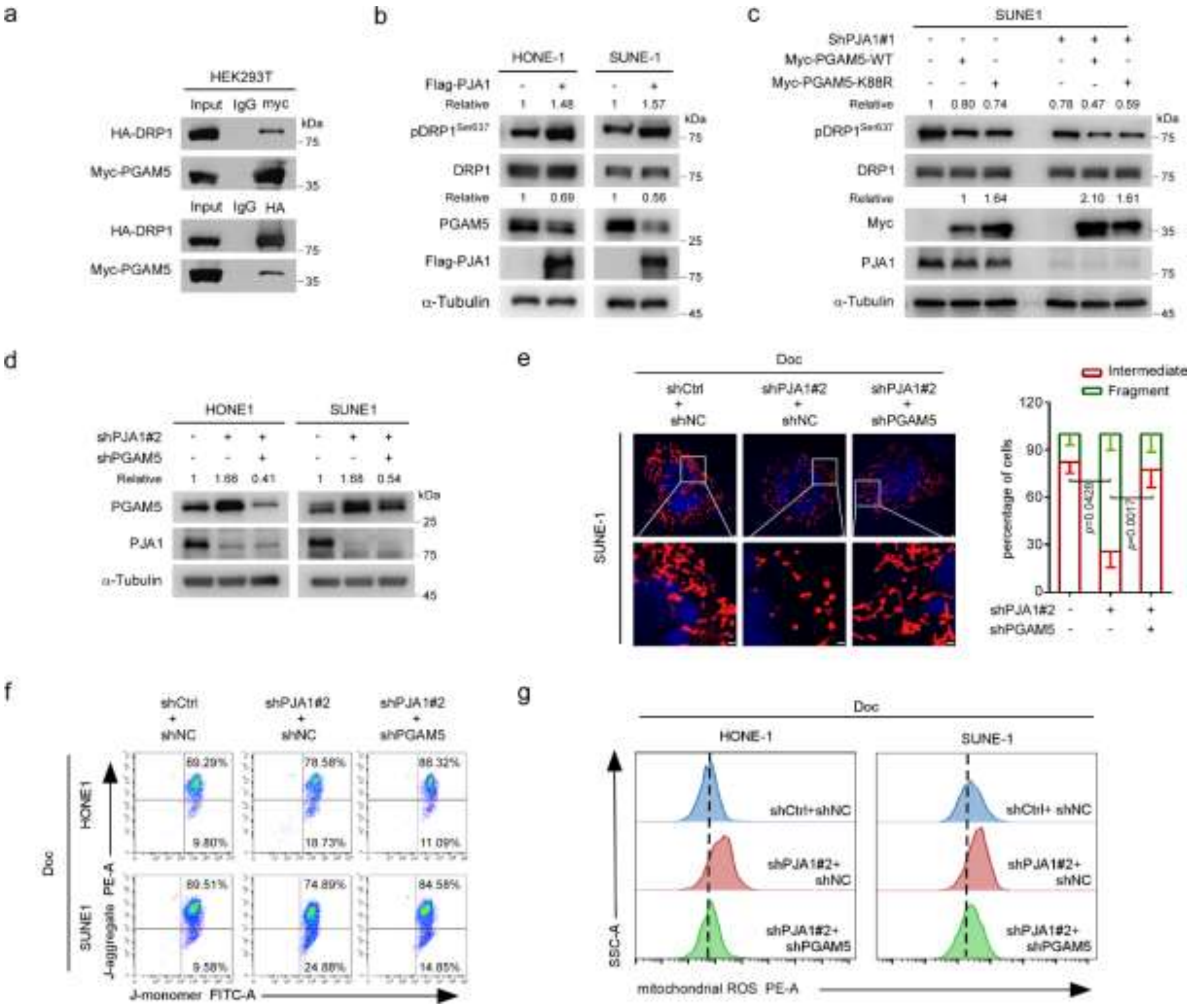

Supplementary Fig. 4 (Extended data related to Main Fig. 4)

a, Co-IP with an anti-Myc (top) or anti-HA (bottom) antibody revealed the exogenous association of DRP1 and PGAM5 in HEK293T cells. **b,** Protein levels of total DRP and pDRP1^{ser637} in NPC cells transfected with the empty vector or Flag-PJA1 plasmids. **c**, Protein levels of total DRP and pDRP1^{ser637} in SUNE1 cells transfected with the shCtrl or sh-PJA1 plasmids together with the Myc-PGAM5-WT or the K88R mutant plasmids. **d,** Protein expression of PJA1 and PGAM5 in NPC cells transfected with the shCtrl or sh-PJA1 plasmids together with the shNC or sh-PGAM5 plasmids. **e,** Representative images of mitochondria in SUNE1 cells transfected with the shCtrl or sh-PJA1 plasmids together with shNC or sh-PGAM5 plasmids, and exposed to docetaxel (Doc, 10 nM). Scale bar, 1 μm. More than 50 cells were counted to determine the proportions of tubular and fragmented mitochondria (mean ± s.d., one-way ANOVA). **f-g,** Representative images of flow cytometric analysis of the mitochondrial membrane potential (**f**) and the production of mROS (**g**) in NPC cells transfected with the shCtrl or sh-PJA1 plasmids together with shNC or sh-PGAM5 plasmids, and exposed to Doc (10 nM). N=3 (e) repeats from three independent experiments. Source data are provided as a Source Data file.

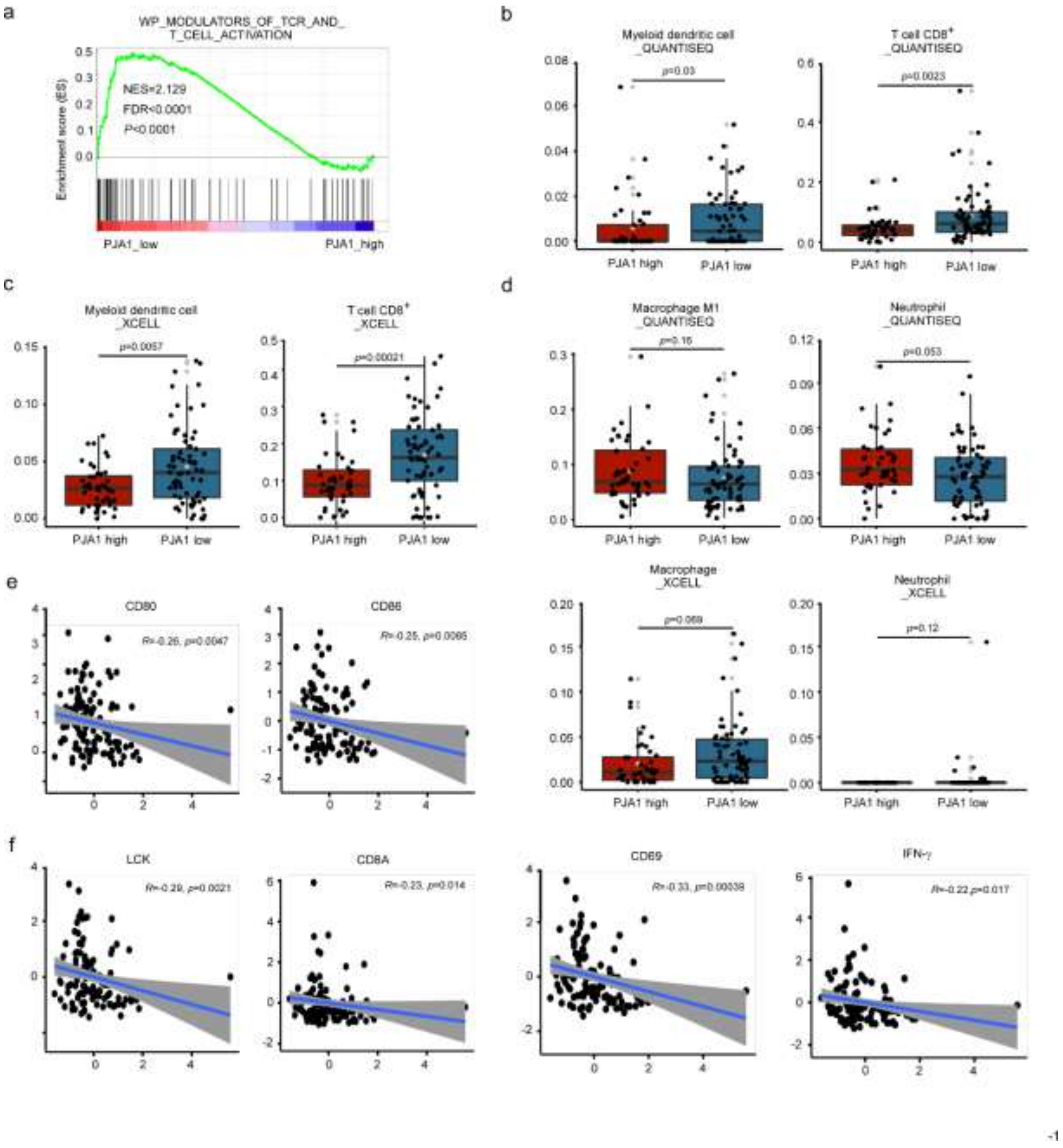

Supplementary Fig. 5 (Extended data related to Main Fig. 6)

a, Gene set enrichment analysis (GSEA)40 based on the GSE102349[40] dataset showing gene sets related to T-cell activation enriched in PJA1-low NPC tissues. **b-c,** QUANTISEQ (**b**) and XCELL (**c**) analyses of dendritic cells (DCs) and CD8$^+$ T cell infiltration into tumours with high or low PJA1 expression (two-tailed unpaired t test). **d,** QUANTISEQ and XCELL analyses of macrophage and neutrophil infiltration into tumours with high or low PJA1 expression (two-tailed unpaired t test). **e-f,** Spearman correlation analysis of PJA1 expression with CD80, CD86, IFNγ, LCK, CD8A and CD69 expression.

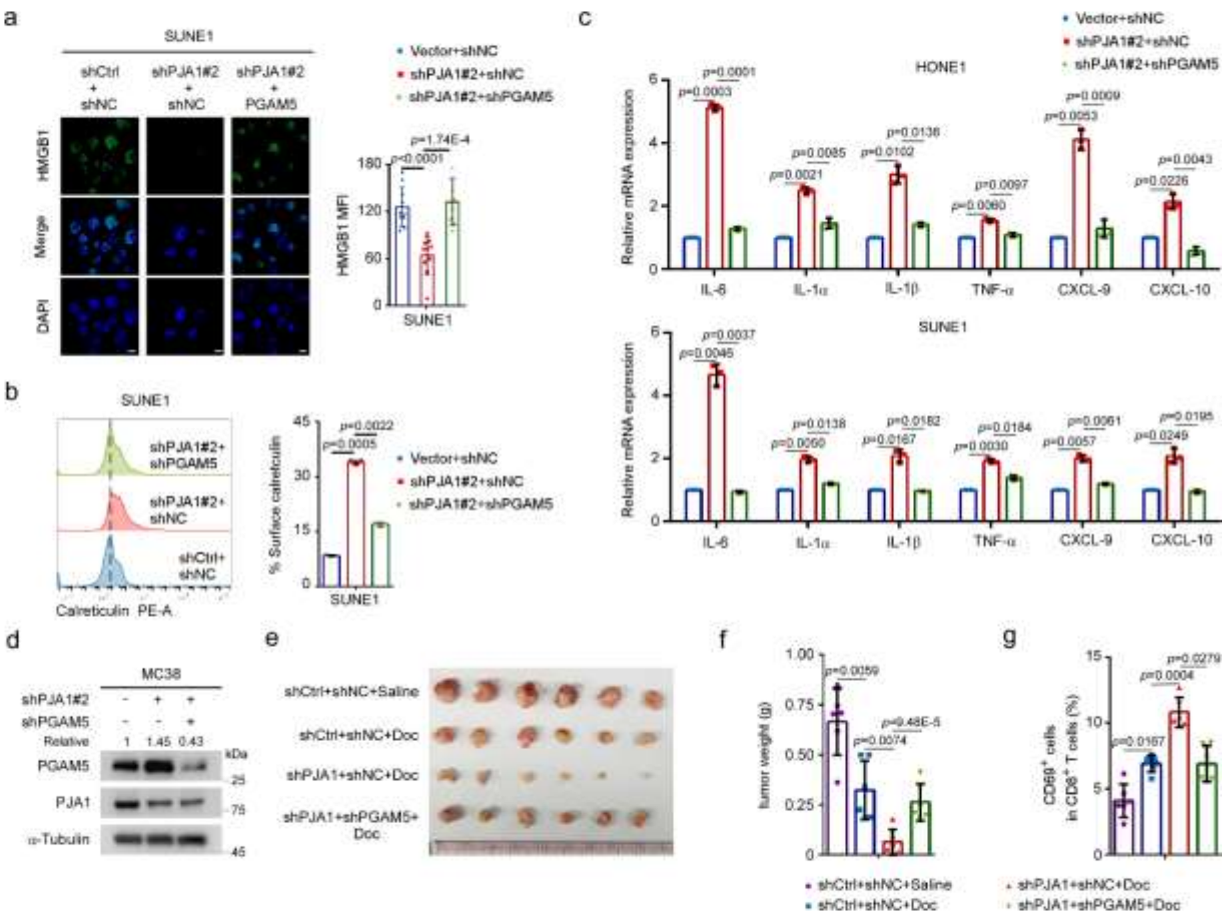

Supplementary Fig. 6 (Extended data related to Main Fig. 6)

a-c, NPC cells stably transfected with the shCtrl or sh-PJA1 plasmids together with shNC or sh-PGAM5 plasmids were treated with docetaxel (Doc, 10 nM) for 48 h. Representative fluorescence images showing the release of HMGB1 from the nucleus into the cytoplasm in SUNE1 cells. Quantitative data from 10 randomly selected fields per group are reported. Scale bar, 20 μm (**a**, mean(n=10) ± s.d., one-way ANOVA). The expression level of calreticulin on the surface of SUNE1 cells was evaluated by flow cytometry (**b**, left: representative plots, right: percentages, mean ± s.d., one-way ANOVA). The mRNA levels of IL-6, IL-1α, IL-1β, TNF-α, CXCL-9 and CXCL-10 in NPC cells (**c**, mean ± s.d., one-way ANOVA). **d,** PJA1 and PGAM5 expression in MC38 cells stably transfected with the shCtrl or sh-PJA1 plasmids together with shNC or sh-PGAM5 plasmids. **e-g,** Macroscopic images (**e**), weights (**f**) and percentages of CD69+CD8+ cells (**g**) of excised MC38 cell-derived tumours (mean (n = 6) ± s.d., one-way ANOVA). N=3 (b) and n=3 (c) repeats from three independent experiments. Source data are provided as a Source Data file.

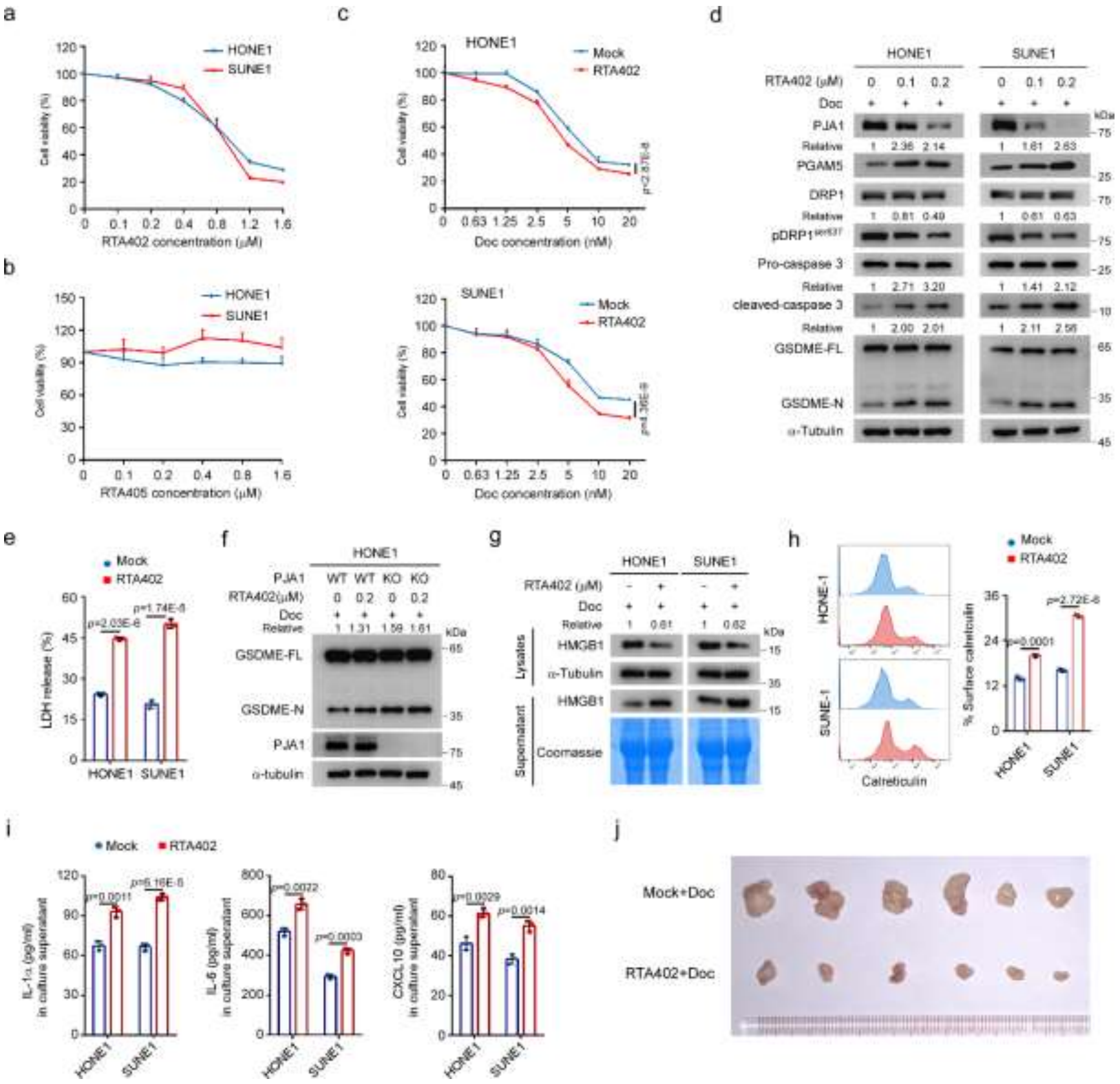

Supplementary Fig. 7 (Extended data related to Main Fig. 7)

a-b, CCK8 assay measuring the sensitivity of NPC cells exposed to the indicated concentrations of RTA402 (**a**) and RTA405 (**b**) (mean ± s.d., two-way ANOVA). **c,** CCK8 assay measuring the sensitivity of NPC cells exposed to the indicated concentration of docetaxel (Doc) together with or without RTA402 (mean ± s.d., two-way ANOVA). **d,** Protein levels of PJA1, PGAM5, total DRP1, pDRP1^{ser637}, and the cleavage of caspase-3 and GSDME in NPC cells treated with or without RTA402. **e,** LDH release from NPC cells treated with or without RTA402 (mean ± s.d., two-tailed unpaired t test). **f,** GSDME cleavage in PJA1-WT and PJA1-KO HONE1 cells exposed to Doc (10 nM) and treated with or without RTA402. **g,** Levels of HMGB1 in the supernatant and lysates of NPC cells treated with or without RTA402. Coomassie staining was used as a control to verify equal gel loading. **h,** The expression level of calreticulin on the surface of NPC cells treated with or without RTA402 was evaluated by flow cytometry (left, representative plots; right, percentages; mean ± s.d., two-tailed unpaired t test). **i,** The concentrations of IL-1α, IL-6 and CXCL10 in the supernatants from NPC cells treated with or without RTA402 were measured by ELISA (mean ± s.d., two-tailed unpaired t test). **j,** Macroscopic images of the excised tumours from humanized NSG mice in each group. N=4 (a, b, c) and n=3 (e, h, i) repeats from three independent experiments. Source data are provided as a Source Data file.

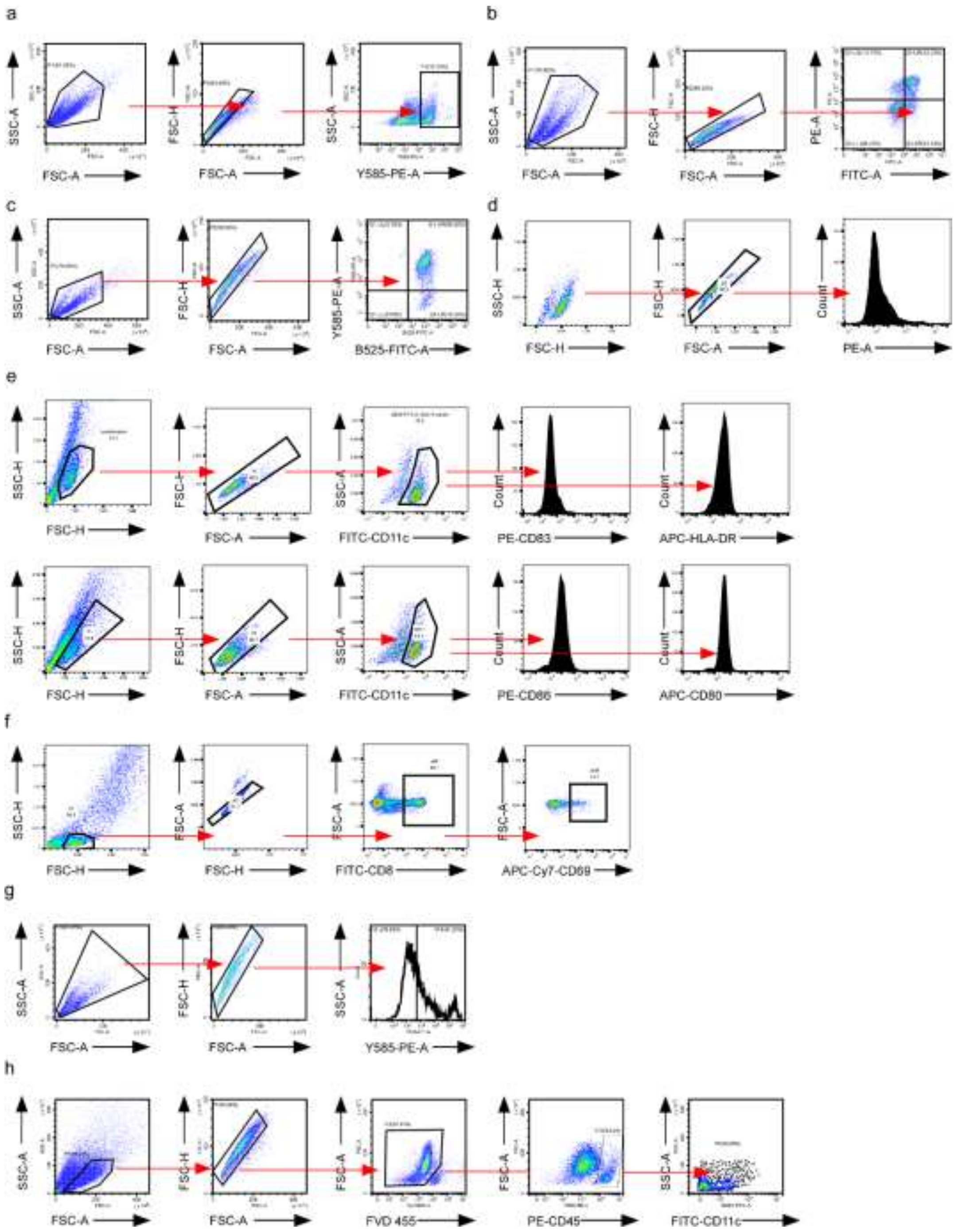

Supplementary Fig. 8 (Gating strategy for flow cytometric analysis)

a, Gating strategy for the flow cytometric analysis in Fig. 1f, Fig. 5f and Supplementary Fig. 2h.
b, Gating strategy for the flow cytometric analysis in Fig. 1m and Fig. 5d.
c, Gating strategy for the flow cytometric analysis in Fig. 4g, Fig. 4l and Supplementary Fig. 4f.
d, Gating strategy for the flow cytometric analysis in Fig. 4i, Fig. 4m and Supplementary Fig. 4g.
e, Gating strategy for the flow cytometric analysis in Fig. 6d.
f, Gating strategy for the flow cytometric analysis in Fig. 6e.
g, Gating strategy for the flow cytometric analysis in Fig. 6h, Supplementary Fig. 6b and Supplementary Fig. 7h.
h, Gating strategy for the flow cytometric analysis in Fig. 6l.

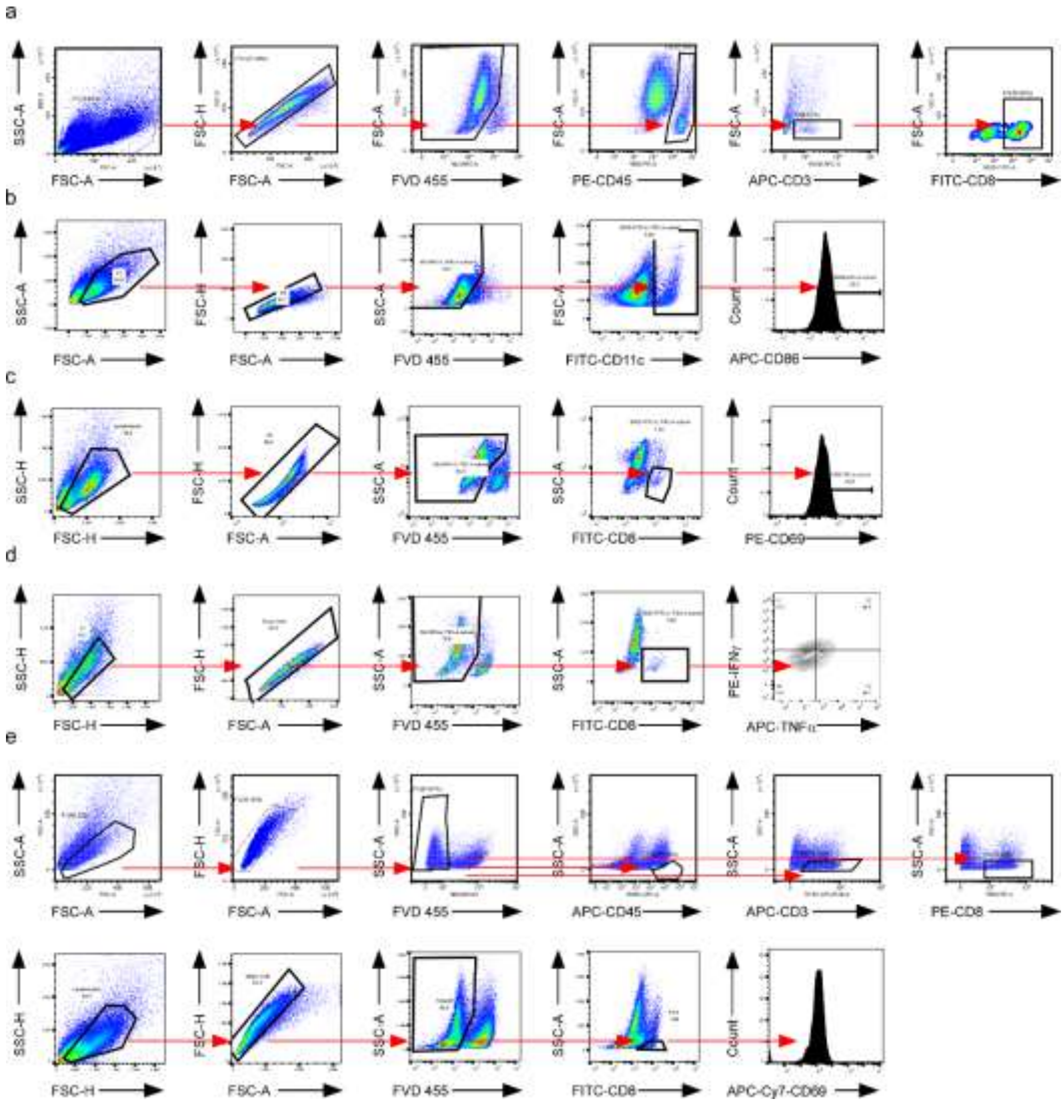

Supplementary Fig.8 (Gating strategy for flow cytometric analysis)

a, Gating strategy for the flow cytometric analysis in Fig. 6l.
b, Gating strategy for the flow cytometric analysis in Fig. 6m.
c, Gating strategy for the flow cytometric analysis in Supplementary Fig. 6g.
d, Gating strategy for the flow cytometric analysis in Fig. 6n.
e, Gating strategy for the flow cytometric analysis in Fig. 7i.

Supplementary Table 1. Mass spectrometry analysis results for the ubiquitination site in the anti-Flag (Flag-tagged PGAM5) immunoprecipitants.

Confidence	Annotated Sequence	Quality PEP	Quality q-value	Master Protein Accessions	Modifications in Master Proteins
High	[R].TLSDYNIQKESTLHLVLR.[L]	6.60E-04	1.00E-03	P62987	P62987 1×GG [K63]
High	[K].LAETTDKVK.[Q]	9.10E-02	6.10E-03	Q06124	Q06124 1×GG [K242]
High	[R].VIFSGSLDFFSDSFFNSAVQK.[A]	1.20E-01	7.10E-03	P39656	P39656 1×GG [K275]
**High**	**[R].NVESGEEELASKLDHYK.[A]**	**1.00E-02**	**1.00E-03**	**Q96HS1**	**Q96HS1 1×GG [K88]**

Supplementary Table 2. Baseline characteristics of nasopharyngeal carcinoma patients with low or high PJA1 expression.

Characteristic	Low expression group n=139	High expression group n=140	P value†
**Age**			
≤ 45	81 (58.3)	78 (55.7)	0.666
> 45	58 (41.7)	62 (44.3)	
**Gender**			
Female	32 (23.0)	42 (30.0)	0.187
Male	107 (77.0)	98 (70.0)	
**WHO type**			
Undifferentiated non-keratinizing	138 (99.3)	138 (98.6)	1.000
Differentiated non-keratinizing	1 (0.7)	2 (1.4)	
**T category***			
T1	3(2.2)	2(1.4)	
T2	11(7.9)	12 (8.6)	0.984
T3	74(53.2)	80(57.1)	
T4	51 (36.7)	46 (32.9)	
**N category***			
N0	5 (3.6)	10 (7.1)	0.103
N1	78 (56.1)	60 (42.9)	
N2	48 (34.5)	54 (38.6)	
N3	8 (5.8)	16 (11.4)	
**TNM stage**			
III	80(57.6)	81(57.9)	0.959
IV	59(42.4)	59(42.1)	
**Treatment**			
IC+CCRT	68 (48.9)	76 (54.3)	0.370
CCRT	71 (51.1)	64 (45.7)	

Notes:

†P value was calculated using the Chi-square tests or Fisher's exact test.

Abbreviations: WHO: World Health Organization. IC: induction chemotherapy, CCRT: concurrent chemotherapy

Supplementary Table 3. Primer sequences of the shRNAs, siRNA and RT–qPCR used in the study.

	Sequence (forward: 5' to 3')
Human shRNAs	
PJA1#1-F	CCGGGCGAGGAGTGACCAAGTGAAACTCGAGTTTCACTTGGTCACTCCTCGCTTTTTG
PJA1#1-R	AATTCAAAAAGCGAGGAGTGACCAAGTGAAACTCGAGTTTCACTTGGTCACTCCTCGC
PJA1#2-F	CCGGCCAATCAGTCAGTTAGCCTAACTCGAGTTAGGCTAACTGACTGATTGGTTTTTG
PJA1#2-R	AATTCAAAAACCAATCAGTCAGTTAGCCTAACTCGAGTTAGGCTAACTGACTGATTGG
PGAM5-F	CCGGGCACGAGATCTTCATCTGTCACTCGAGTGACAGATGAAGATCTCGTGCTTTTTG
PGAM5-R	AATTCAAAAAGCACGAGATCTTCATCTGTCACTCGAGTGACAGATGAAGATCTCGTGC
Human siRNA	
PGAM5	ACGAGATCTTCATCTGTCA
Mouse shRNAs	
PJA1-F	CCGGCGACGATTACTACCGATACTACTCGAGTAGTATCGGTAGTAATCGTCGTTTTTG
PJA1-R	AATTCAAAAACGACGATTACTACCGATACTACTCGAGTAGTATCGGTAGTAATCGTCG
PGAM5-F	CCGGCTGGAGAAGACGAGTTGACATCTCGAGATGTCAACTCGTCTTCTCCAGTTTTTG
PGAM5-R	AATTCAAAAACTGGAGAAGACGAGTTGACATCTCGAGATGTCAACTCGTCTTCTCCAG
RT–qPCR	
GAPDH-F	GTCTCCTCTGACTTCAACAGCG
GAPDH-R	ACCACCCTGTTGCTGTAGCCAA
PJA1-F	GGATACCGCCAATGACAATGA
PJA1-R	CGGGATAGCCACTTGAACTCTC
PGAM5-F	ATCTGTCACGCCAACGTCATCC
PGAM5-R	CAGCAAGTGAAAGAGGTCAGGAC
CXCL10-F	GGTGAGAAGAGATGTCTGAATCC
CXCL10-R	GTCCATCCTTGGAAGCACTGCA
CXCL9-F	CTGTTCCTGCATCAGCACCAAC
CXCL9-R	TGAACTCCATTCTTCAGTGTAGCA
IL-1α-F	TGTATGTGACTGCCCAAGATGAAG
IL-1α-R	AGAGGAGGTTGGTCTCACTACC
IL-1β-F	CCACAGACCTTCCAGGAGAATG
IL-1β-R	GTGCAGTTCAGTGATCGTACAGG
TNF-α-F	CTCTTCTGCCTGCTGCACTTTG
TNF-α-R	ATGGGCTACAGGCTTGTCACTC
IL-6-F	AGACAGCCACTCACCTCTTCAG
IL-6-R	TTCTGCCAGTGCCTCTTTGCTG

Supplementary Table 4. List of antibodies used in Western blotting and Immunoprecipitation in this study.

Western blotting					
**Primary antibodies**	**Supplier**	**Catalog Number**	**Source**	**Species activity**	**dilution ratio**
anti-PJA1	Proteintech	17687-1-AP	Rabbit	Hu, Mo	1:400
anti-PGAM5	Abcam	ab126534	Rabbit	Hu	1:2000
anti-DRP1	Cell Signaling Technology	8570S	Rabbit	Hu, Mo	1:1000
anti-DFNA5/GSDME	Abcam	ab215191	Rabbit	Hu, Mo	1:1000
anti-Caspase-3	Cell Signaling Technology	9915T	Rabbit	Hu, Mo	1:2000
anti-Cleaved Caspase-3	Abcam	ab32042	Rabbit	Hu	1:1000
anti-Cleaved Caspase-3	Cell Signaling Technology	9915T	Rabbit	Hu, Mo	1:2000
Anti-HMGB1	Abcam	ab79823	Rabbit	Hu, Mo	1:20000
anti-DRP1 Ser637	Invitrogen	PA5-37534	Rabbit	Hu, Mo	1:500
anti-FALG	Sigma–Aldrich	F1804	Mouse		1:500
anti-Flag	Sigma–Aldrich	F3165	Mouse		1:500
anti-HA	Sigma–Aldrich	H6908	Rabbit		1:1000
anti-Myc	Proteintech	16286-1-AP	Rabbit		1:2000
anti-GAPDH	Sigma–Aldrich	G8795	Mouse	Hu, Mo	1:5000
anti-α-Tubulin	Proteintech	66031-1-Ig	Mouse	Hu, Mo	1:1000
**Secondary antibodies**					
anti-rabbit	Cell Signaling Technology	7074	Goat	rabbit	1:5000
anti-mouse	Cell Signaling Technology	7076	Horse	mouse	1:5000
Immunoprecipitation					
anti-HA	Sigma–Aldrich	H6908	Rabbit		3μg
anti-Flag	Sigma–Aldrich	F1804	Mouse		3μg
anti-Myc	Proteintech	16286-1-AP	Rabbit		3μg
Normal Mouse IgG	Invitrogen	10400C	Mouse		3μg
Normal Rabbit IgG	Invitrogen	10500C	Rabbit		3μg
anti-PJA1	Proteintech	17687-1-AP	Rabbit	Hu, Mo	3μg
anti-DRP1	Cell Signaling Technology	8570S	Rabbit	Hu, Mo	3μg

Supplementary Table 5. List of antibodies used in Immunofluorescence (IF) , Immunohistochemical (IHC) staining and Flow cytometric analysis in this study.

IF					
**Primary antibodies**					
anti-DNM1L（DRP1）	Invitrogen	MA5-26255	Mouse	Hu, Mo	1:100
anti-Flag	Sigma–Aldrich	F1804	Mouse		1:40
anti-PGAM5	Abcam	ab126534	Rabbit		1:100
**Secondary antibodies**					
Alexa Fluor 488	Invitrogen	A11008	Goat	Rabbit	1:1000
Alexa Fluor 647	Invitrogen	A32728	Goat	Mouse	1:1000
IHC					
anti-CD11c	Abcam	ab52632	Rabbit	Hu	1:100
anti-CD8	Abcam	ab4055	Rabbit	Hu	1:100
anti-CD3	Abcam	ab11089	Rabbit	Hu	1:100
anti-PJA1	Invitrogen	PA5-51419	Rabbit	Hu	1:50
Flow cytometric analysis					
**Primary antibodies**	**Supplier**	**Catalogue No.**	**Clone**	**Species Specificity**	**Application**
FITC–anti-human CD11c	Biolegend	337214	Bu15	Hu	FC
APC–anti-human HLA-DR	Biolegend	307610	L243	Hu	FC
APC–anti-human CD80	Biolegend	305220	2D10	Hu	FC
PE–anti-human CD83	Biolegend	305308	HB15e	Hu	FC
PE–anti-human CD86	Biolegend	374206	BU63	Hu	FC
FITC–anti-human CD8a	Biolegend	300906	HIT8a	Hu	FC
APC/Cyanine7–anti-human 69	Biolegend	310914	FN50	Hu	FC
APC/Cyanine7–anti-human CD3	Biolegend	300425	UCHT1	Hu	FC
APC–anti-human CD45	Biolegend	304011	HI30	Hu	FC
PE-anti-human CD8					
PE–anti-mouse CD45	Biolegend	103105	30-F11	Mouse	FC
FITC–anti-mouse CD8a	Biolegend	100706	53-6.7	Mouse	FC
APC-anti-mouse CD3	Biolegend	100235	17A2	Mouse	FC
FITC–anti-mouse CD11c	Biolegend	117305	N418	Mouse	FC
APC-anti-mouse CD86	Biolegend	105011	GL-1	Mouse	FC
PE-anti-mouse CD69	Biolegend	104508	H1.2F3	Mouse	FC
APC–anti-human/mouse Granzyme B recombinant	Biolegend	396407	QA18A28	Mouse	FC
PE–anti-mouse IFNγ	Biolegend	505807	XMG1.2	Mouse	FC
APC–anti-mouse TNFα	Biolegend	506308	MP6-XT22	Mouse	FC
eBioscience™ Fixable Viability Dye eFluor™ 455UV	Thermo Fisher Scientific	65-0868-14			
