# Supplementary material for: PJA1-mediated suppression of pyroptosis as a driver of docetaxel resistance in nasopharyngeal carcinoma
Source: Nat Commun. 2024 Jun 21;15:5300. doi: 10.1038/s41467-024-49675-2 (PMC11192944; doi:10.1038/s41467-024-49675-2)
Supplement: Supplementary file 2 — Reporting Summary [file 41467_2024_49675_MOESM2_ESM.pdf]

nature portfolio

Corresponding author(s): NCOMMS-23-46075

Last updated by author(s): Jun 12, 2024

# Reporting Summary

Nature Portfolio wishes to improve the reproducibility of the work that we publish. This form provides structure for consistency and transparency in reporting. For further information on Nature Portfolio policies, see our Editorial Policies and the Editorial Policy Checklist.

## Statistics

For all statistical analyses, confirm that the following items are present in the figure legend, table legend, main text, or Methods section.

| n/a | Confirmed | |
|---|---|---|
| ☐ | ☒ | The exact sample size (*n*) for each experimental group/condition, given as a discrete number and unit of measurement |
| ☐ | ☒ | A statement on whether measurements were taken from distinct samples or whether the same sample was measured repeatedly |
| ☐ | ☒ | The statistical test(s) used AND whether they are one- or two-sided<br>*Only common tests should be described solely by name; describe more complex techniques in the Methods section.* |
| ☐ | ☒ | A description of all covariates tested |
| ☐ | ☒ | A description of any assumptions or corrections, such as tests of normality and adjustment for multiple comparisons |
| ☐ | ☒ | A full description of the statistical parameters including central tendency (e.g. means) or other basic estimates (e.g. regression coefficient) AND variation (e.g. standard deviation) or associated estimates of uncertainty (e.g. confidence intervals) |
| ☐ | ☒ | For null hypothesis testing, the test statistic (e.g. *F*, *t*, *r*) with confidence intervals, effect sizes, degrees of freedom and *P* value noted<br>*Give P values as exact values whenever suitable.* |
| ☒ | ☐ | For Bayesian analysis, information on the choice of priors and Markov chain Monte Carlo settings |
| ☒ | ☐ | For hierarchical and complex designs, identification of the appropriate level for tests and full reporting of outcomes |
| ☐ | ☒ | Estimates of effect sizes (e.g. Cohen's *d*, Pearson's *r*), indicating how they were calculated |

*Our web collection on statistics for biologists contains articles on many of the points above.*

## Software and code

Policy information about availability of computer code

| | |
|---|---|
| Data collection | No software was used. |
| Data analysis | No software was used. |

For manuscripts utilizing custom algorithms or software that are central to the research but not yet described in published literature, software must be made available to editors and reviewers. We strongly encourage code deposition in a community repository (e.g. GitHub). See the Nature Portfolio guidelines for submitting code & software for further information.

## Data

Policy information about availability of data

All manuscripts must include a data availability statement. This statement should provide the following information, where applicable:

- Accession codes, unique identifiers, or web links for publicly available datasets
- A description of any restrictions on data availability
- For clinical datasets or third party data, please ensure that the statement adheres to our policy

The mRNA expression profiling datasets ( (GSE132112, Gene Expression Omnibus (GEO) repository, https://www.ncbi.nlm.nih.gov/geo/query/acc.cgi?acc=GSE132112), (GSE102349, Gene Expression Omnibus (GEO) repository, https://www.ncbi.nlm.nih.gov/geo/query/acc.cgi?acc=GSE102349)) in this study were previously published (1. Lei Y, et al. A Gene-Expression Predictor for Efficacy of Induction Chemotherapy in Locoregionally Advanced Nasopharyngeal Carcinoma. J Natl Cancer Inst. 2021 Apr 6;113(4):471-480. doi: 10.1093/jnci/djaa100. 2. Zhang L, et al. Genomic Analysis of Nasopharyngeal Carcinoma Reveals TME-Based Subtypes. Mol Cancer Res. 2017 Dec;15(12):1722-1732. doi: 10.1158/1541-7786.MCR-17-0134.). The statistical source data for Figs. 1–7 and Supplementary Figs.

1–7 is provided in the Source Data file, and two mass spectrometry analyses are provided in Supplementary Table 1 and Supplementary data 1. The unprocessed images of the immunoblot bands are displayed in Source Data file . The raw data generated in this study including CCK8, qPCR, western blotting, flow cytometric analysis, immunofluorescence staining, animal experiments, and so on, have been deposited in the Research Data Deposit of Sun Yat-sen University Cancer Center with an accession number RDDB2024700296 (https://www.researchdata.org.cn).The raw data are available under restricted access for reproducing the findings, access can be obtained from the corresponding author. The raw data are protected and are not available due to data privacy laws. The processed data generated in this study are provided in the Source Data file.

## Research involving human participants, their data, or biological material

Policy information about studies with human participants or human data. See also policy information about sex, gender (identity/presentation), and sexual orientation and race, ethnicity and racism.

| | |
|---|---|
| Reporting on sex and gender | The gender information of 279 patients with nasopharyngeal carcinoma were retrospectively collected through the medical record. We reported the gender distribution of 279 NPC patients in the Supplementary Table 3. |
| Reporting on race, ethnicity, or other socially relevant groupings | No report on race, ethnicity, or other socially relevant groupings were conducted in this study. |
| Population characteristics | The clinical characteristics of NPC patients are shown in Supplementary Table 3. |
| Recruitment | We collected 50 fresh-frozen NPC samples for expression analysis and collected 279 paraffin-embedded NPC samples from the Sun Yat-sen University Cancer Center (Guangzhou, China) between between 2009 and 2016 for survival analysis. |
| Ethics oversight | This study was approved by the Institutional Ethical Review Boards of Sun Yat-sen University Cancer Center, and the requirement for informed consent from each patient was exempted (G2021-026-01). |

Note that full information on the approval of the study protocol must also be provided in the manuscript.

# Field-specific reporting

Please select the one below that is the best fit for your research. If you are not sure, read the appropriate sections before making your selection.

[x] Life sciences [ ] Behavioural & social sciences [ ] Ecological, evolutionary & environmental sciences

For a reference copy of the document with all sections, see nature.com/documents/nr-reporting-summary-flat.pdf

# Life sciences study design

All studies must disclose on these points even when the disclosure is negative.

| | |
|---|---|
| Sample size | We collected 50 fresh frozen and 279 paraffin-embedded NPC specimens between 2009 and 2016 at Sun Yat-sen University Cancer Center (Guangzhou, China) |
| Data exclusions | No data were exluded from the analyses. |
| Replication | All attempts at replications were successful. |
| Randomization | The samples used in this study were randomly assigned into control or experimental groups. |
| Blinding | The investigators were not blinded to sample allocation, because results used were obtained using objective quantitative methods. |

# Reporting for specific materials, systems and methods

We require information from authors about some types of materials, experimental systems and methods used in many studies. Here, indicate whether each material, system or method listed is relevant to your study. If you are not sure if a list item applies to your research, read the appropriate section before selecting a response.

## Materials & experimental systems

| n/a | Involved in the study | |
|---|---|---|
| [ ] | [x] | Antibodies |
| [ ] | [x] | Eukaryotic cell lines |
| [x] | [ ] | Palaeontology and archaeology |
| [ ] | [x] | Animals and other organisms |
| [ ] | [x] | Clinical data |
| [x] | [ ] | Dual use research of concern |
| [x] | [ ] | Plants |

## Methods

| n/a | Involved in the study | |
|---|---|---|
| [x] | [ ] | ChIP-seq |
| [ ] | [x] | Flow cytometry |
| [x] | [ ] | MRI-based neuroimaging |

## Antibodies

| | |
|---|---|
| Antibodies used | The antibodies used are shown in Supplementary Table 5. |
| Validation | All antibodies were validated by Western blotting, immunofluorescence imaging or IHC staining prior to isotope-polymer conjugation. Antibodies were tested for cell type and inter-cell location specificity within positive control tissues. |

## Eukaryotic cell lines

Policy information about cell lines and Sex and Gender in Research

| | |
|---|---|
| Cell line source(s) | The human NPC cell lines HONE1 and SUNE1 were generously supplied by Professor Musheng Zeng from Sun Yat-sen University Cancer Center. HEK293T cells and MC38 murine colon adenocarcinoma cells were obtained from American Type Culture Collection (ATCC). |
| Authentication | None of the cell lines were authenticated. |
| Mycoplasma contamination | All the cells were cultured for less than 2 months, and tested for mycoplasma contamination. |
| Commonly misidentified lines (See ICLAC register) | None. |

## Animals and other research organisms

Policy information about studies involving animals; ARRIVE guidelines recommended for reporting animal research, and Sex and Gender in Research

| | |
|---|---|
| Laboratory animals | Female BALB/c nude mice and C57BL/6 mice (4~5 weeks old) were purchased from Charles River Laboratories (Zhejiang). Female SPF humanized NSG mice (6~8 weeks old) were purchased from Shanghai Model Organisms Center, Inc. (Shanghai), and the percentages of human CD45+ cells in the peripheral blood of these mice were determined to be greater than 1% one week after tail vein injection of human PBMCs (5×106). |
| Wild animals | The study did not involve wild animals. |
| Reporting on sex | All animals used in this study were female, and no sex-based analysis was performed. |
| Field-collected samples | The study did not involve field-collected samples. |
| Ethics oversight | All animal experiments were approved by the Experimental Animal Ethics Committee of Sun Yat-sen University Cancer Center (L025501202108011). |

Note that full information on the approval of the study protocol must also be provided in the manuscript.

## Clinical data

Policy information about clinical studies

All manuscripts should comply with the ICMJEguidelines for publication of clinical research and a completedCONSORT checklist must be included with all submissions.

| | |
|---|---|
| Clinical trial registration | This study did not report data from clinical trials. |
| Study protocol | 279 paraffin-embedded NPC specimens collected between 2009 and 2016 at Sun Yat-sen University Cancer Center (Guangzhou, China) were a part of a clinical trial (TPF IC). Another 50 fresh frozen tissue samples were obtained from NPC patients receiving standard treatment. All patients were restaged according to the 7th edition of American Joint Committee on Cancer staging manual. |
| Data collection | We collected the patients' clinical characteristics including age, gender,the WHO pathological type, tumour stages, and the administration of TPF induction chemotherapy, as well as the survival data including overall survival time, disease-free survival time and distant metastasis-free survival time. |
| Outcomes | The primary endpoint was disease-free survival (DFS), and the secondary endpoints were distant metastasis-free survival (DMFS) and overall survival (OS). We defined DFS as the period from the first date of treatment to the date of disease progression or death from any cause, whichever occurred first; DMFS as the time to the occurrence of distant metastasis of the disease; OS as the time from the first day of treatment to the date of death from any cause . |

## Plants

| | |
|---|---|
| Seed stocks | The study did not involve any plants. |
| Novel plant genotypes | The study did not involve any plants. |
| Authentication | The study did not involve any plants. |

## Flow Cytometry

### Plots

Confirm that:

- [x] The axis labels state the marker and fluorochrome used (e.g. CD4-FITC).
- [x] The axis scales are clearly visible. Include numbers along axes only for bottom left plot of group (a 'group' is an analysis of identical markers).
- [x] All plots are contour plots with outliers or pseudocolor plots.
- [x] A numerical value for number of cells or percentage (with statistics) is provided.

### Methodology

| | |
|---|---|
| Sample preparation | The sample preparation was described in the methods section. |
| Instrument | All data were obtained with a CYTOFLEX flow cytometer (Beckman Coulter). |
| Software | The results were analysed using CYTOFLEX flow cytometer or Flow Jo software 10. |
| Cell population abundance | In each analysis, minimum of 5,000 cells were counted. |
| Gating strategy | The gating strategy were shown in the Supplementary Figure 9. |

- [x] Tick this box to confirm that a figure exemplifying the gating strategy is provided in the Supplementary Information.
