# Supplementary material for: PJA1-mediated suppression of pyroptosis as a driver of docetaxel resistance in nasopharyngeal carcinoma
Source: Nat Commun. 2024 Jun 21;15:5300. doi: 10.1038/s41467-024-49675-2 (PMC11192944; doi:10.1038/s41467-024-49675-2)
Supplement: Supplementary file 3 — Description of Additional Supplementary Files [file 41467_2024_49675_MOESM3_ESM.pdf]

Description of Additional Files

Supplementary Data 1: Mass spectrometry analysis results for the anti-Flag (Flag-tagged PJA1) immunoprecipitants.
